# Editorial: Sustainable horticulture: from omic sciences to new breeding techniques

**DOI:** 10.3389/fpls.2023.1257469

**Published:** 2023-08-09

**Authors:** Sara Sestili, Jaime Prohens, Nadia Ficcadenti, Romina Beleggia

**Affiliations:** ^1^ Council for Agricultural Research and Economics (CREA) Research Centre for Vegetable and Ornamental Crops, Monsampolo del Tronto (AP), Italy; ^2^ Instituto de Conservacion y Mejora de la Agrodiversidad Valenciana, Universitat Politècnica de València, Valencia, Spain; ^3^ Council for Agricultural Research and Economics (CREA) Research Center for Cereals and Industrial Crops, Foggia (FG), Italy

**Keywords:** genetic resources, genome editing, fruit quality, water use efficiency, metabolic profile

## Editorial on the Research Topic

The rapidly growing world population and the changing climate are increasing the food demand so much that transitioning to more sustainable horticulture has become one of the main challenges in recent years. Several strategies can enhance sustainability in horticulture, including adopting good agricultural practices (GAP) to improve soil fertility, optimizing water and nitrogen use efficiencies, and implementing low-input management techniques to preserve the environment. In this scenario, horticultural plant breeding is mainly focused on developing varieties adapted to specific environmental conditions and agronomic techniques. These varieties aim to provide higher yields and nutritional values that meet the demands while minimizing the use of resources, contributing to more biodiverse horticulture.

The improvement of important agronomic and quality traits for sustainable horticulture can be obtained by using several approaches, ranging from the high-throughput sequencing technologies in the field of the omics sciences (e.g. genomics, transcriptomics, proteomics, metabolomics, and phenomics) and the new breeding techniques (NBT), to the conservation, enhancement, and the use of plant genetic resources (PGR) with unique organoleptic and functional characteristics (Enfissi et al., 2021; Mahmood et al., 2022; Shen et al., 2022).

The Solanaceae family is one of the most important worldwide in terms of the number of species, economic importance and has a widespread distribution (in tropical, subtropical, and temperate regions). It is extensively utilized for human consumption, making it crucial to prioritize sustainable practices within the family The Research Topic “*Sustainable horticulture: from omics sciences to new breeding techniques*” features three papers that focus on using plant genetic resources to identify genes related to fruit quality in different Solanaceae species. Additionally, it includes a paper exploring the use of magnetized water as an innovative green technology in diverse crops (durum wheat and lentil).

In the case of eggplant (*Solanum melongena*), a study investigated the presence of chlorophyll in fruit peel and its influence on both fruit color and nutritional composition (Arrones et al.). The dissection of relevant traits has been carried out by using F2 and BC1 mapping populations. However, the advent of multi-parental populations has improved the mapping of quantitative trait loci (QTLs) and the identification of candidate genes related to traits of interest in several species. A multi-parent advanced generation inter-cross (MAGIC) population was employed to identify major candidate genes controlling the fruit skin chlorophyll biosynthesis in eggplant. A genome-wide association study (GWAS) indicated the gene APRR2 (ARABIDOPSIS PSEUDO RESPONSE REGULATOR2) is the principal responsible for fruit chlorophyll in the peel. The APRR2 gene has been validated on the G2P-SOL eggplant germplasm core collection and phylogenetic studies contributed to understanding APRR2 evolution among different species (Arrones et al.).

Highlighting the significance of genetic resources, another paper emphasized the immense potential of tomato heirlooms (*Solanum lycopersicum* L.) for genetic improvement and candidate gene identification (Tripodi et al.). Local or traditional tomato varieties are well adapted to a particular cultivation area where they have been selected over the years by farmers considering quality characteristics and regional market preferences as selection criteria. In this scenario, heirlooms are of great importance both for being a reservoir of genes conferring resistance to biotic and abiotic stress and for being the representation of the local culture. Tripodi et al. studied 60 tomato heirlooms belonging to the Beefsteak, Cherry, Globe, Oxheart, and Plum types, at the phenotypic, genomic, and biochemical levels and identified genes related to important fruit quality characteristics. The biochemical characterization revealed that the selection performed by farmers was focused mainly on morphology (fruit weight) and fruit color (β-carotene). The availability of the SolCAP Tomato Infinium array allowed the authors to perform phylogenetic studies to shed light on the relationships among the heirlooms and to validate the metabolic and genomic data obtained. The accessions were distinguished by the typologies, with plum and cherry types clearly distinguished from beefsteak and globe ones, as well as by fruit sizes and shapes. Furthermore, the genes SLSUN31 and CCD3 are involved in the fruit weight increase and the conversion of all-*trans*-β-carotene to strigolactones, respectively. These results underscored the significance of the heirloom tomato varieties not only for their fruit quality characteristics but also because they represent a valid material for candidate gene discovery for traits such as resistance to biotic and abiotic stresses.

In tomatoes, the most abundant phenolic compound is chlorogenic acid (CGA). This metabolite possesses antioxidant and antimicrobial activities, which confers resistance to some diseases and protects against UV-B light. D’Orso et al. used CRISPR technology to generate knock-out mutant lines in the HQT gene to assess its effect on caffeoylquinic acids (CQAs) biosynthesis as the only relevant metabolic route and evaluated the physiological roles of the CGA produced and accumulated mainly regarding the protection against UV-B irradiation. The results demonstrated the complete absence of CGA or other caffeoylquinic acids (CQAs) in edited plants clarifying the role of HQT in the biosynthesis of most abundant phenolic compounds in Solanaceae. Moreover, the serious damage shown by CGA-lacking plants after UV exposure confirms the pivotal role of this metabolite in protecting against UV-B irradiation. It also revealed a reorganization of the phenylpropanoid metabolism involving both the redirection of flux to other biochemical branches and the remodeling of the expression of important regulators (D’Orso et al.).

Finally, an innovative approach to contribute to sustainable horticulture and plant growth improvement is the use of magnetized water for irrigation. Sestili et al. demonstrated the positive effects of this practice on the development of durum wheat and lentil plantlets (roots and epicotyls). Although the results varied depending on the species, tissues, and time points considered, compared with tap water (TW), the magnetized water treatment (MWT) led to higher root elongation in both genotypes while no effect was recorded in the epicotyl length. Significant variations were reported also in the metabolic composition. Amino acids in roots and sugars in epicotyls were mainly positively affected by MWT with a higher or even “new” correlation with the growth parameters compared to the control. The study showed that in durum wheat, sugar metabolism was mainly influenced, while in lentil, other than sucrose, organic acids are mostly affected, highlighting the species-specific response to MWT and their correlation with plant development. The results suggest that magnetized water use in agriculture can be considered a sustainable technology that promotes plant development and improves quality while reducing water usage, leading to cost savings and environmental protection.

Although the Research Topic collected few articles, these contributions advance the understanding of agronomic and quality traits. Furthermore, the introduction of innovative technology, such as the use of magnetized irrigation water, offers promising prospects for sustainable agriculture and the production of healthier food.

## Author contributions

SS: Writing – original draft. JP: Writing – review & editing. NF: Writing – review & editing. RB: Writing – original draft.
